# Twelfth Annual Commencement of the Southern Medical College

**Published:** 1891-03

**Authors:** 


### TWELFTH ANNUAL COMMENCEMENT OF THE SOUTHERN MEDICAL COLLEGE.

---

The twelfth annual commencement of the Southern Medical College was held at DeGive's opera house yesterday afternoon.

On the stage sat the members of the college faculty and the gentlemen whose names were down for participation in the programme. The dean of the faculty, Dr. William Perrin Nicolson, presided. The exercises were opened with prayer by the Rev. Walker Lewis, of Trinity church.

Dr. Nicolson, as dean, then read his annual report as follows:

At the close of the twelfth session of the Southern Medical College, I have to report that there has been much progress towards the realization of the purposes of the founders, in making this a school whose tendency should bc to elevate the standard of medical teaching in the South, and during the session now closing, it is a pleasure to note that the standing of the men in the school has been of a nature very encouraging to our hopes in that direction.

There has been in attendance upon the two departments 207 students, representing almost every state in this country, and in the medical department alone there have been in actual attendance 102 students.

The policy of this schocl has never been the securing of a large class independent of the material of which it is composed, and the greater cheapness of many competitors, and the more easy attainment of a degree in medicine, have a tendency always to lessen the number but increase the efficiency of the matriculants who do come. It is along this line that we have followed for the past twelve years, and we see no reason why we should abandon this policy for the sole purpose of filling our benches. In spite of the acknowledged more rigid requirements, and the maintenance of rates of tuition, we have this session instructed twenty more men than last, and the rapid increase can only be attributed to the fact that our efforts are being appreciated.

It gives us much pleasure to state that the increasing number in the medical and dental school renders necessary the erection of a new and commodious building, steps towards which are now being taken, and we hope at the coming session to open the course in quarters more in keeping with the demands upon us.

Dr. Nicolson then presentnd the graduates with their diplomas.

W. B. Barton, W. C. Bateman, M. A. Born, O. S. Clyatt, W. P. Cofer, C. W. Cossey, Robert L. Davis, Alexander Dawson, W. K. Eason, W. R. Edwards, Albert Fensch, C. H. Finley, T. H. Fritts, J. T. Gainmage, J. G. Going, H. L. Ison, E. C. Jeter, C. E. Johnson, J. H. Johnson, T. S. Layton, J. H. McCullough, G. W. McDowell, J. MacDiarmid, J. W. Price, C. J. Ramsey,

D. C. Rumph, J. F. F. Scarborough, C. W. Sheppard, W. H. Stimson, J. P. Sudderth, T. H. Thrasher, A. H. Tickell, W. B. Vaughan, J. M. D. Wall, F. G. Williams, W. S. Wood.

The venerable Dr. Thomas S. Powell conferring the degrees. Dr. Powell then took occasion to speak a few parting words to his children, as he called the young students.

"It gives me much pleasure," he said, "to feel that, by zealous application to the study of your chosen profession, you have honorably won your diplomas in the Southern Medical College. In accordance with the laws of Georgia you are now doctors of medicine, and in the name of the trustees and faculty I congratulate you and give you the benediction of your alma mater. In the very beginning of your professional life I would have you faithfully observe the ethics and rules of the profession, but remember that the truly great physician not only labors continually to reach the acme of medical science so as to prevent as well as to relieve physical ills, but he has the heart of gold that goes out in compassion, and brotherly love and sympathy, ready to minister without partiality or prejudice to all who need his services—the rich and the poor, the learned and the ignorant, the great and the low.

"In bidding you farewell, I would thank you for the courtesy and kindness shown to your instructors, and the assiduous attention given to your studies. May the benediction of heaven go with you to your homes, and rest upon you in all your life's career."

The valedictorian of the class was Dr. McDiarmid, of Georgia. He made a splendid address.

Mr. Charles A. Read, the well-known young lawyer, delivered the annual address to the graduating class.

Then the prizes were distributed. And Dr. T. H. Fritts, of Tennessee, was called as the winner of the first prize, which was a beautiful gold medal given for the best average of the class.

Dr. D. C. Rumph, of Texas, was awarded the second prize, a valuable case of surgical instruments, for the second best general average.

The third prize, a case of pocket instruments, was awarded to Dr. J. W. Price, of Virginia.

Dr. J. W. McDiarmid, of Georgia, won the demonstrator of anatomy's prize, a handsome case of instruments.

The exercizes were closed with prayer by Rev. Mr. Lewis.

